# Energy Dissipated in Fatigue and Creep Conditions

**DOI:** 10.3390/ma14164724

**Published:** 2021-08-21

**Authors:** Stanisław Mroziński, Zbigniew Lis, Halina Egner

**Affiliations:** 1 Faculty of Mechanical Engineering, UTP University of Science and Technology, Al. Prof. Kaliskiego 7, 85-796 Bydgoszcz, Poland; stmpkm@utp.edu.pl (S.M.); Zbigniew.Lis@utp.edu.pl (Z.L.); 2 Faculty of Mechanical Engineering, Cracow University of Technology, Al. Jana Pawła II 37, 31-864 Kraków, Poland

**Keywords:** low-cycle fatigue, creep, strain energy

## Abstract

The paper presents the results of tests performed on samples made of P91 steel under combined variable and constant load conditions, at temperature *T* = 600 °C. The analysis of the test results was carried out with the use of the energetic description of the fatigue process. It was shown that the order of occurrence of the fatigue load and creep in the load program influences the fatigue life and the value of the energy cumulated in the sample until fracture.

## 1. Introduction

The analysis of the mechanical conditions of many structural machine elements shows that they are most often cyclic loads. This applies, inter alia, to facilities operating at elevated temperatures. The design and construction process of elements subject to thermo-mechanical fatigue is performed under the assumption that the selected materials do not contain any initial damage. Moreover, it is assumed that no failure will occur during their service life [1,2]. However, such facilities as power plants, power units, pressure vessels, thermal installations, suffer from failure of their constituent structural elements which need to be replaced. For this and other reasons, the variable load is often stopped, while the constant load is maintained, causing creep, which is not taken into account in the design process. Many types of failure have been observed in engineering practice, such as force-induced elastic deformation, plasticization, buckling, fatigue, cracking, corrosion, thermal shock, etc. [3]. Creep is also listed as one of the possible fatigue damage modes because cyclic creep of the material changes the nature of the load and the durability of the objects. In such a case, predicting the fatigue life based on the commonly used fatigue characteristics may lead to divergence between the experimental and predicted results [4]. In works [5,6], based on constant-amplitude tests, it was shown that the fatigue life of the samples under the conditions of constant stress amplitude ($\sigma_{a}=const$) is lower than that obtained in the conditions of controlled deformation ($\varepsilon_{at}=const$). Creep was given as the reason for the reduction in durability under stress-controlled conditions. For this reason, intensive work is underway to develop a durability prediction method that will fully take into account the physical phenomena accompanying the fatigue process [7,8]. 

Currently, the stress- and strain-based descriptions are still widely used when calculating fatigue life. However, researchers increasingly refer to energy-based approaches to explain the phenomena taking place during cyclic loading [9,10]. Such a description not only combines the stress- and strain-based quantities but also takes into account the mutual interactions between them. The basis for energy descriptions of the fatigue process is most often the strain energy dissipated in the material during cyclic loading until the element fails [11,12,13,14]. Failure is determined by the critical value of the energy dissipated in the material until the crack occurs. 

Assessment of the fatigue life of structural elements is inextricably linked with the summation of fatigue damage. Therefore, it is natural that the physical quantity used to describe fatigue is expected to be measurable and additive. Such a quantity is e.g., the plastic strain energy dissipated during the fatigue test. Taking energy as a criterion quantity to describe fatigue damage allows for assigning damage to each load cycle, the measure of which may be, for example, the hysteresis loop area. Such a property creates a wide range of possibilities for formulating new hypotheses of damage summation, in which the criterion value is the energy accumulated until the crack occurs. The energy description allows for a simple connection of the level of fatigue damage with the energy dissipated during the tests and the number of cycles.

The energy-based approach to the description of fatigue seems to better reflect the physical mechanisms of the fatigue phenomenon and may be used to develop more effective algorithms of fatigue life estimation, especially under the conditions of simultaneous occurrence of constant and cyclic loads.

## 2. Materials and Methods

### 2.1. Energy and Damage

The critical value of energy dissipated during the fatigue test in the range of cyclic elasto-plastic strains can be calculated by summing up the areas of all hysteresis loops, $W_{cr}=W_{pl}\left(N\right)=\sum_{i=1}^{N}\Delta W_{pl}^{}\left(i\right)$. The calculations very often apply the simplifying assumption that the loop area does not change during the fatigue process. This means that the total energy dissipated during the test can be determined by multiplying the energy $\Delta {\tilde{W}}_{pl}$, dissipated in one cycle of the material stabilization period (or, in the case of non-stabilizing materials, from half-life 0.5N), by the number of cycles to fracture N:

(1) $$W_{pl}\left(N\right)=N\cdot \Delta {\tilde{W}}_{pl}$$

There are many energy-based models of fatigue, in which, apart from the plastic strain energy, other energies (total energy, elastic strain energy) are taken into account (see [15]). In the following work, attention is focused on the description of low-cycle fatigue, in which only the plastic strain energy is taken into account.

Based on the research on composites and steels presented in papers [16,17] it was shown that it is possible to analytically describe the relationship between the energy dissipated in the sample until its fracture, $W_{pl}\left(N\right)$, and the number of cycles to fracture N, using the function of the form:

(2) $$W_{pl}\left(N\right)=\alpha_{\Delta W}lgN+K_{\Delta W}$$

in which $\alpha_{\Delta W}$ and $K_{\Delta W}$ are coefficients.

In [16] the above formula was used as the basic relation for summing the fatigue damage in terms of the plastic strain energy, while in papers [18,19]—for the analysis of the low-cycle fatigue tests results obtained under various load conditions. The energy-based description can also be used when analyzing the results of tests performed under the conditions of load programs including a sequence of constant (creep) and variable (fatigue) loads. The deformation energy during creep lasting for period τ, $W_{c}\left(\tau \right)$, can be calculated as follows [8]:

(3) $$W_{c}\left(\tau \right)=\sigma_{c}\cdot \varepsilon_{c}\left(\tau \right)$$

where $\sigma_{c}$ denotes constant stress acting during creep time τ, and $\varepsilon_{c}\left(\tau \right)$ is the respective creep strain. Total deformation energy, $W_{t}$, dissipated during the load sequence containing both variable and constant loads, will be the sum of the plastic strain energy $W_{f}\left(n\right)$, dissipated during n cycles of the fatigue test, and energy $W_{c}\left(\tau \right)$ dissipated during creep test period τ, which can be written as [7]:

(4) $$W_{t}=W_{f}\left(n\right)+W_{c}\left(\tau \right)=\overset{n}{\underset{i=1}{\sum }}\Delta W_{pl}^{}\left(i\right)+\sigma_{c}\cdot \varepsilon_{c}\left(\tau \right)$$

Currently, there are several hypotheses of fatigue damage accumulation under the conditions of simultaneous cyclic and constant loads. They have been discussed extensively, for example in [7]. A linear model refers directly to the proposal described in [18]. According to this approach, after the implementation of k periods of cyclic loading consisting of $n_{i}$ cycles, and l periods of creep loading lasting time $\tau_{i}$, total damage level $D_{t}$ will be the sum of damage caused by the cyclic load, $D_{f}$, and creep, $D_{c}$:

(5) $$D_{t}=D_{f}+D_{c}=\overset{k}{\underset{i=1}{\sum }}\frac{n_{i}}{N}+\overset{l}{\underset{i=1}{\sum }}\frac{\tau_{i}}{T_{c}}$$

where N is the number of cycles to failure under fatigue conditions only ($\sigma_{a}=const$ and τ=0), while $T_{c}$ stands for the time to crack occurrence in the creep test only ($\sigma_{c}=const$, n=0).

Both components of total damage $D_{t}$ in Equation (5) can be presented using the strain energy dissipated in the fatigue test and the strain energy dissipated in the creep test. Relation (5) will then take the following form:

(6) $$D_{t}=D_{f}+D_{c}=\overset{k}{\underset{i=1}{\sum }}\frac{W_{f}\left(n_{i}\right)}{W_{f}\left(N\right)}+\overset{l}{\underset{i=1}{\sum }}\frac{W_{c}(\tau_{i})}{W_{c}\left(T_{c}\right)}$$

**Figure 1:**
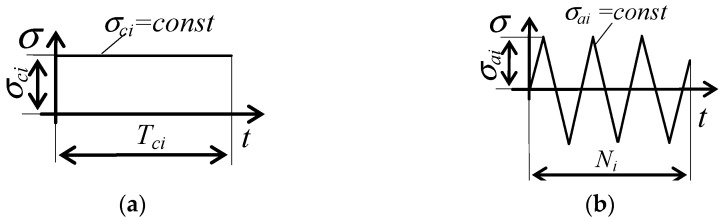

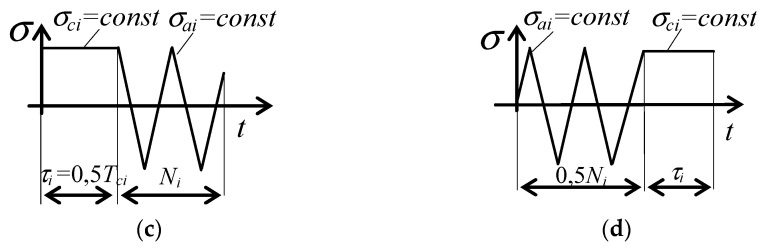
Schemes of load programs: (**a**) creep (c); (**b**) fatigue (f); (**c**) creep then fatigue (c+f); (**d**) fatigue then creep (f + c). The following load levels were applied: $\sigma_{c1}=\sigma_{a1}=219\;\mathrm{MPa}$, $\sigma_{c2}=\sigma_{a2}=225\;\mathrm{MPa}$, $\sigma_{c3}=\sigma_{a3}=240\;\mathrm{MPa}$, $\sigma_{c4}=\sigma_{a4}=249\;\mathrm{MPa}$, $\sigma_{c5}=\sigma_{a5}=258\;\mathrm{MPa}$.

It should be emphasized that both approaches, described by Equations (5) and (6), are insensitive to the order of occurrence of particular load types and, therefore, ignore the history of changes in the microstructure of the material under various load conditions. The research problem undertaken in the present work was to investigate the effect of a creep load and its sequence in the load scheme, on the fatigue life and the amount of energy accumulated to crack, under the conditions of alternating fatigue and creep conditions (see Figure 1).

### 2.2. Experimental Testing

**Figure 2:**
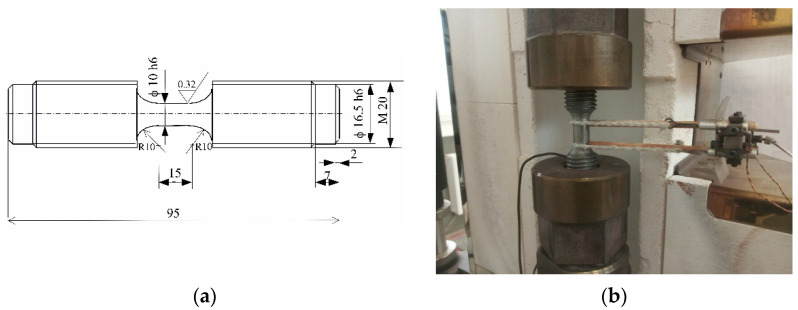
(**a**) Test sample geometry; (**b**) test stand.

Samples for tests were prepared from P91 steel ($R_{m}$ = 353 MPa, $R_{p0.2}$ = 564 MPa, $A_{5}$ = 63.3%, E = 129.7 GPa at the temperature of 600 °C) and shaped following the guidelines specified in the standard [20]. The dimensions of samples are shown in Figure 2a. Tests included various load configurations (only creep, only fatigue with R = −1, and tests in which the samples were subjected to alternating fatigue and creep loads in various order, see Figure 1). The changes in the load type were made after half of the cycles to failure were completed under the conditions of fatigue (0.5*N*), or after the time of 0.5$T_{c}$ under creep conditions. The tests were carried out at the temperature *T* = 600 °C. 

Constant load (creep) tests and fatigue tests were performed on an Instron 8502 testing machine equipped with a heating chamber with a maximum temperature range of $T_{max}$ = 1000 °C. The stand for static and fatigue tests is shown in Figure 2b. The temperature of the sample was monitored with a thermocouple attached to the grip part of the sample (Figure 2b). The heating speed was 10 °C/min. A sample, mounted in the holders of the testing machine, was not subjected to mechanical loads during heating. Creep tests were carried out under the guidelines provided in [21]. Tests (constant or variable loads) were always started at about 10 min after stabilizing the sample temperature to 600 °C. The temperature of a sample during the fatigue tests (program f) and creep tests (program c) was maintained automatically by the heating chamber control system. During the change of the type of load (programs f-c and c-f, see Figure 1), the mounting method and the temperature of samples remained unchanged. After completion of the tests, samples were cooled along with the chamber.

The sample deformation was measured with an extensometer with a measurement base of 12.5 mm. The frequency of cyclic loads was 0.2 Hz. During the constant load (creep) as well as the variable load (fatigue), the instantaneous values of the force loading the sample as well as its deformation were recorded.

## 3. Results

### 3.1. Constant Load Tests (Creep)

**Figure 3:**
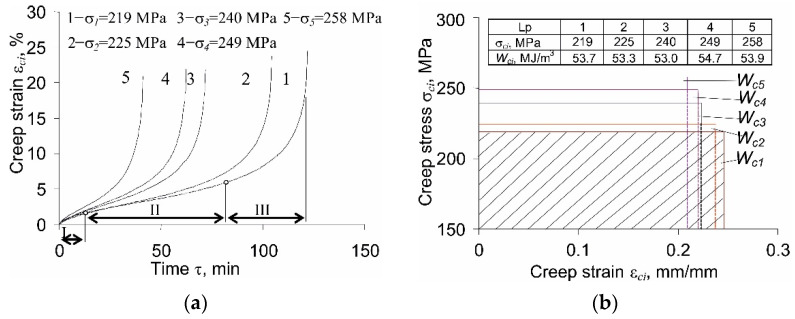
Creep results: (**a**) creep strain *ε_c_* = *f*(*τ*); (**b**) creep stress vs. creep strain *σ_c_* = *f*(*ε_c_*), illustration of creep energy.

As expected, the sample elongation in time (creep) was observed under constant load conditions ($\sigma_{c}=const$). Figure 3a shows examples of creep strains of the sample $\varepsilon_{c}$ at five stress levels (see Figure 1), while Figure 3b presents the results of creep tests in the coordinate system ($\varepsilon_{c},\sigma_{c}$). The calculation of creep energy $W_{c}$ was performed with the use of relation (3).

In all creep diagrams (Figure 3a) up to the instant of fracture, three characteristic stages with different rates of elongation of the specimen in time are visible. These stages have been marked on the diagram obtained for the lowest stress ($\sigma_{c1}$ = 219 MPa): stage I (decreasing strain rate), stage II (constant strain rate), and stage III (increasing strain rate). The stress level $\sigma_{ci}$ affects both the duration of these stages and the elongation rates in individual stages. Based on Figure 3a, it can be concluded that the magnitude of the strain at fracture slightly decreases with increasing stress. In the case of creep strain energy dissipated until failure, $W_{ci}$ (Figure 3b), the influence of the stress level on its value is even less pronounced; the values of creep strain energy at the five stress levels $\sigma_{ci}$ are very similar. Small differences in creep strain energy at failure confirm that the assumption of constant creep energy as a creep failure condition is correct [6,8,22,23].

### 3.2. Fatigue Tests

**Figure 4:**
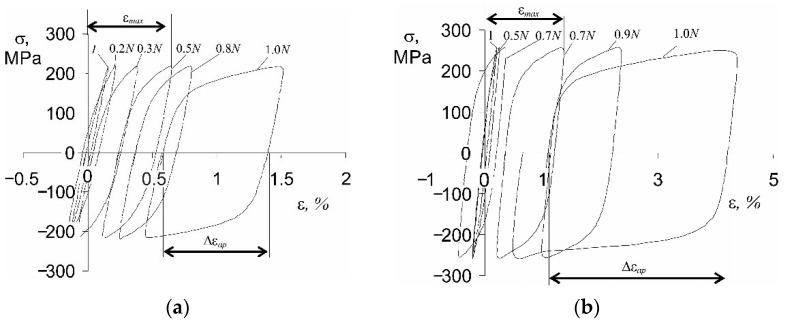
Chosen hysteresis loops at two stress levels: (**a**) $\sigma_{a1}$ = 219 MPa, (**b**) $\sigma_{a5}$ = 258 MPa.

Instantaneous values of the loading force and deformation of the sample recorded during the tests were used for the analysis of the hysteresis loop parameters. Figure 4 shows examples of hysteresis loops at two stress levels: $\sigma_{a1}$ = 219 MPa and $\sigma_{a5}$ = 258 MPa (see Figure 1).

**Figure 5:**
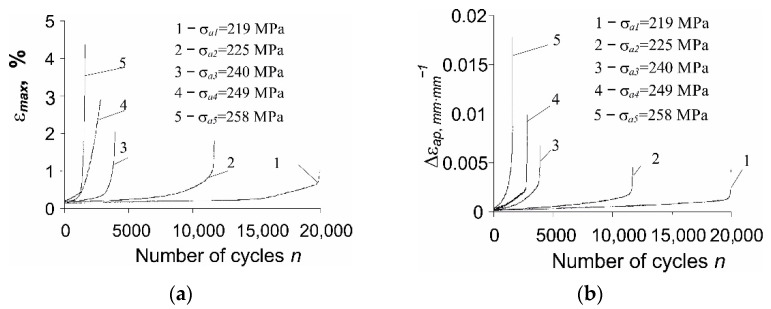
Changes of loop parameters during cyclic load: (**a**) *ε_max_*; (**b**) Δ*ε_ap_*.

As expected, during fatigue loads under the conditions of controlled stress $\sigma_{a}=const$, cyclic creep of the material is observed as an increase in the subsequent maximum loop strain $\varepsilon_{max}$. The evolution of $\varepsilon_{max}$ in a fatigue test is influenced by the stress amplitude (see Figure 5a). In addition to the increase in the relative elongation $\varepsilon_{max}$ with the growing value of $\sigma_{a}$, the range of loop plastic deformation $\Delta \varepsilon_{ap}$ also increases (Figure 5b). Changes in the above-mentioned hysteresis loop parameters versus the load cycle number are shown in Figure 5a,b.

The shift of the hysteresis loops along the strain axis is a consequence of the asymmetry of strains during the tension–compression half-cycle. While the strain asymmetry was not observed under the conditions of elastic strains, the phenomenon becomes apparent when the yield point is exceeded. When the stress exceeds the yield point, the P91 steel exhibits slightly higher resistance to deformation during compression. Based on the analysis of the loops shown in Figure 4, it can be concluded that with the increase in stress, the deformation asymmetry also increases, which results in the shift of the hysteresis loop. Strain asymmetry during tension and compression was observed and analyzed in many studies [5,6].

The increase in plastic deformation $\Delta \varepsilon_{ap}$ during tension-compression load cycles allows the conclusion that P91 steel belongs to the class of materials exhibiting cyclic softening. This phenomenon was also observed under the conditions of controlled deformation [6].

**Figure 6:**
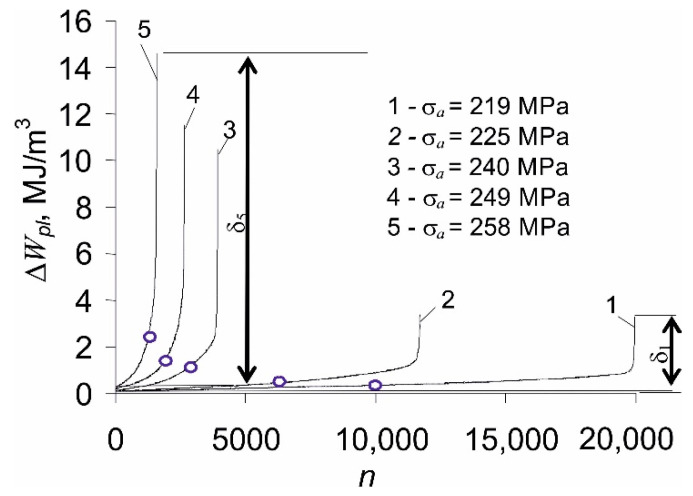
Plastic strain energy in fatigue tests in function of load cycle number, Δ*W_pl_* = *f*(*n*).

The increase in the relative elongation of the sample, $\varepsilon_{max}$, as well as the change in plastic strain, $\Delta \varepsilon_{ap}$, are the result of the loading conditions (stress control, $\sigma_{a}=const$). On the other hand, under the conditions of $\varepsilon_{at}=const$ (strain-controlled tests with total strain amplitude $\varepsilon_{at}$), cyclic creep does not occur. For this reason, strain-controlled tests are preferable, because the existence of creep during stress-controlled tests hinders the analysis of test results [19]. Based on the research described in the literature [5,6], it can be concluded that changes in loop plastic strain $\Delta \varepsilon_{ap}$ under the conditions of $\sigma_{a}=const$ are much larger than changes in this parameter under $\varepsilon_{at}=const$. The consequence of variations in loop plastic strains is the alteration of the plastic strain energy produced in one load cycle, $\Delta W_{pl}$. Based on the recorded instantaneous values of the loading force and deformation of the sample, the energies $\Delta W_{pl}\left(n\right)$ for subsequent load cycles were calculated and are presented in Figure 6.

The increase in plastic strains with the growing number of load cycles, observed in Figure 5b, causes the energy of the hysteresis loop $\Delta W_{pl}$ to change as well. This is because the energy $\Delta W_{pl}$ takes into account the interactions between the parameters of the hysteresis loop, such as Δσ, $\Delta \varepsilon_{ap}$. The amount of energy changes is influenced by the level of the alternating load ($\sigma_{a}$). As the stress amplitude increases, also the changes in loop energy $\Delta W_{pl}$ are larger ($\delta_{5}>\delta_{4}>\cdots >\delta_{1}$). As expected, the loop energy $\Delta W_{pl}$, increases with increasing stress amplitude. 

**Figure 7:**
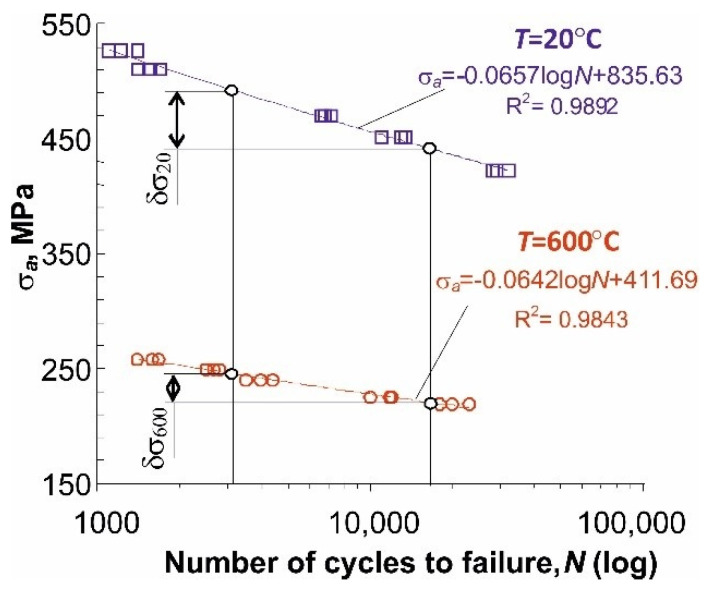
Stress amplitude versus number of cycles to failure, for two test temperatures: 600 °C and 20 °C.

The results of fatigue tests at the temperature of 600 °C under the conditions of $\sigma_{a}=const$ are shown in Figure 7 in the form of a classic fatigue plot in the logN-$\sigma_{a}$ semi-logarithmic coordinate system. To illustrate the influence of temperature on fatigue life, the figure additionally includes the results of fatigue tests of P91 steel samples at room temperature (T = 20 °C). Fatigue plots have been approximated by straight lines with the equations shown in the figure. The analysis of the slope of the fatigue diagrams shows the higher sensitivity of P91 steel to changes in stress at elevated temperature ($\delta \sigma_{600}<\delta \sigma_{20}$). Therefore, slight changes in stress at elevated temperatures may lead to significant changes in durability. For this reason, accurate fatigue life prediction is a key problem for the design of objects operating at elevated temperatures. One should also be aware that the low-cycle fatigue plots in terms of stress are arbitrary due to the presence of significant plastic strains, see Figure 4.

**Figure 8:**
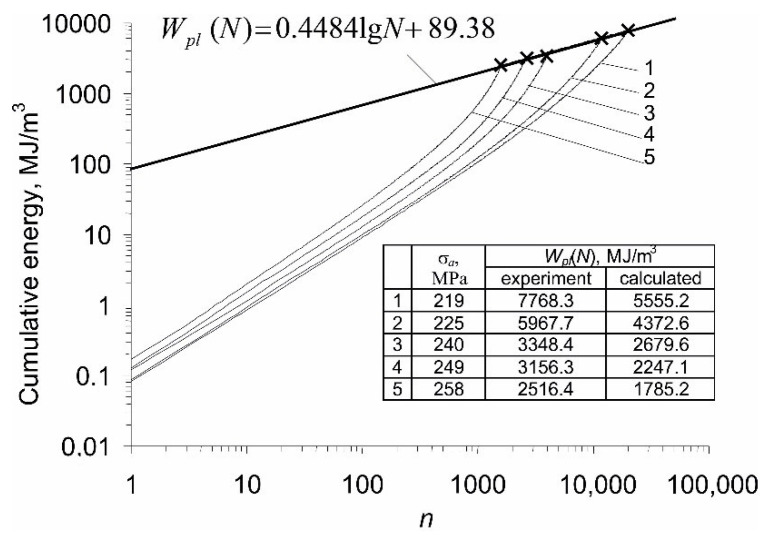
Plastic strain energy accumulation during constant amplitude fatigue tests.

Based on the results obtained from constant amplitude fatigue tests, Figure 8 shows the energy accumulation waveforms at five stress levels.

The values of energy cumulated in the sample until failure, $W_{pl}\left(N\right)$, obtained at individual stress levels, were approximated by a straight line described by Equation (2). The graphs of energy accumulation at individual stress levels are characterized by a clear progression. This is due to the increase in loop energy $\Delta W_{pl}\left(n\right)$, observed in Figure 6. Significant variations in the energy $\Delta W_{pl}\left(n\right)$ raise doubts whether the results obtained with the use of Equation (1) are correct. To illustrate the problem, the table placed in Figure 8 summarizes the critical energy values $W_{pl}\left(N\right)$ at individual stress levels, estimated in two different ways: (1) experimentally, as the sum of the energies of all hysteresis loops, and (2) calculated as the product of the loop energy from half the fatigue life by the number of cycles to failure (Equation (1)). The results presented in the table clearly show that the energy calculated according to Equation (1) is always lower than the energy obtained from tests, with no clear influence of the stress amplitude level on the differences between the experiment and calculations. The differences in the cumulative energy $W_{pl}\left(N\right)$ from calculations and tests confirm that the Formula (1) may be too simplified and does not account for constant changes in cyclic properties of P91 steel, which belongs to the class of materials that do not exhibit a stabilization period. This problem was addressed in the literature many times, for example in works [23,24].

### 3.3. Alternating Creep-Fatigue Tests (c-f)

**Figure 9:**
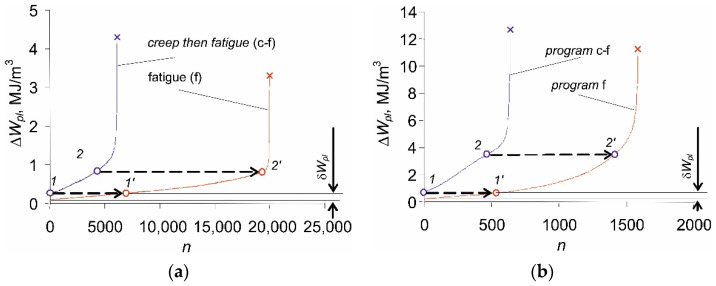
Plastic strain energy versus load cycle for different stress amplitude levels: (**a**) *σ_a_* = 219 MPa; (**b**) *σ_a_* = 247 MPa.

Figure 9 shows plastic strain energy $\Delta W_{pl}$ versus the number of a cycle for two levels of cyclic stress amplitude: the lowest ($\sigma_{a}$ = 219 MPa) and $\sigma_{a}$ = 249 MPa for samples previously subjected to creep during time τ = 0.5$T_{c}$. For comparison, Figure 9 additionally contains the energy functions for a constant-amplitude cyclic load (f) only, performed with the same amplitude levels.

The analysis of the results allows for the conclusion that preceding the cyclic load (*f*) with a constant load (c), apart from reducing the durability, shifts the plastic strain energy $\Delta W_{pl}$ in the first load cycle concerning the load program without permanent loads. When a creep load (in the c-f program) is applied before cyclic loading, the energy of the first hysteresis loop (point 1) corresponds to the hysteresis loop energy in the pure fatigue (f) program, but after a certain number of load cycles was performed (point 1’). It is interesting that only a slight effect of the creep stress level $\sigma_{c}$ on the initial energy shift $\delta W_{pl}$ was observed. At the same time, the level of $\sigma_{c}$ significantly influences “the distance” between plastic strain energy evolution curves of (c-f) and (f) loading programs. Moreover, there is a certain level of the hysteresis loop energy for both (c-f) and (f) cases, which separates the zones of lower and higher energy rates (points 2 and 2’). It is noteworthy that this energy is very similar in both runs. The above may suggest that a certain constant loop energy value is necessary for the initiation of a fatigue crack at a given stress amplitude level, and it seems not to depend on the load history.

**Figure 10:**
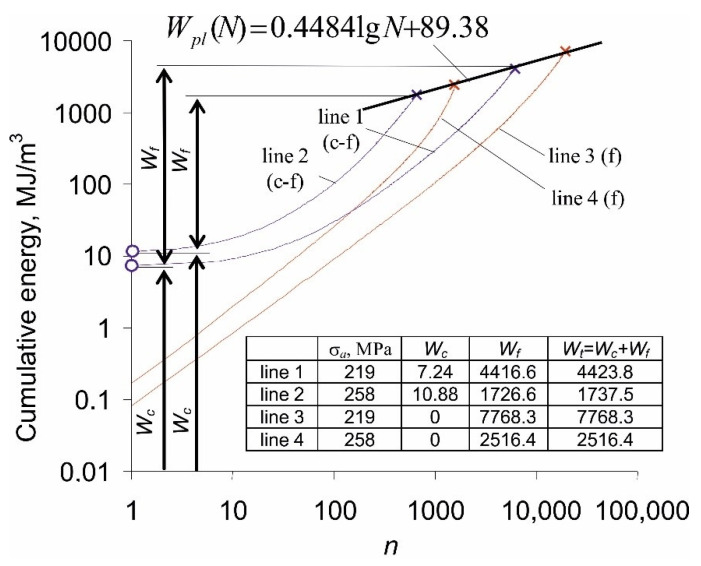
Energy accumulation in the course of (c-f) and (f) tests.

The test results were next used for the analysis of energy accumulation in both (c-f) and (f) load schemes. Figure 10 shows the course of energy accumulation for two stress levels, realized under the conditions of a cyclic load only (f), and a cyclic load preceded by a creep load (c-f). Additionally, the critical value of energy according to Equation (2) is presented in the figure.

Energy accumulation under the (c-f) program conditions starts from the value of the energy dissipated in the creep test ($W_{c}$). Based on the results, it can be concluded that the total values of the energy ($W_{t}=W_{c}+W_{f}$) for the samples under the conditions of programmed loads are very close to the boundary diagram for pure fatigue, resulting from Equation (2) (see also Figure 8).

### 3.4. Alternating Fatigue-Creep Tests (f-c)

**Figure 11:**
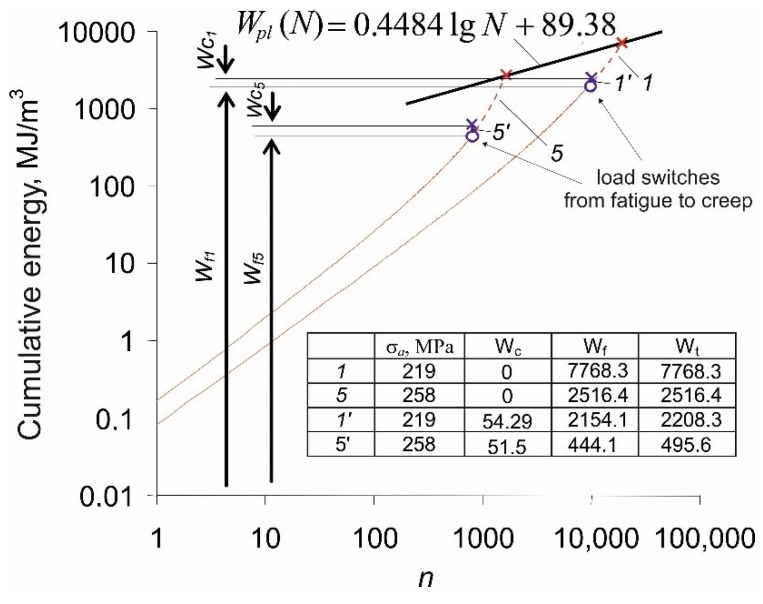
Energy accumulation in the course of (f-c) and (f) tests.

During the implementation of the load variant (f-c), the creep load was preceded by the fatigue load. The loop energy changes for the fatigue part of this test are the same as for the constant-amplitude load (f) (Figure 6). However, the accumulation of energy in the whole (f-c) test is different. The accumulation of plastic strain energy in the (f) part of the test is then followed until failure by the accumulation of creep energy (see Figure 11). The whole curves *1* and *5* correspond to the energy plots in Figure 8. In the points indicated in the figure, the load switches from fatigue to creep, and the cumulative energy paths switch from 1 to 1’, and from 5 to 5’. As a result, the critical values of the total accumulated energy at failure are much lower than in the case of a pure fatigue load test.

In Figure 11 it can be seen that the critical values of the energy accumulated in the sample during the (f-c) program are definitely below the limit line described by Equation (2). The values of energy cumulated in samples during the implementation of two load variants (c-f and f-c, see Figure 1) are compared in Table 1. A pronounced effect of the load sequence on both the critical energy and the total number of cycles is observed.

Based on the above results, it can be concluded that the load sequence has a significant impact on the sample durability. Preceding a fatigue load with a constant load (c-f program) causes a reduction in fatigue life concerning the test in which creep is preceded by fatigue. The load sequence in the program affects also the values of the creep and plastic strain energies dissipated in the sample during the test. In the case of the (c-f) test, the creep strain energy *W_c_* is several times lower than the relevant energy dissipated in the sample during the (f-c) program. The differences in creep strain energies *W_c_* between both load programs are related to different creep strain rates observed in Figure 3. In the case of the (c-f) test, the creep time *τ* = 0.5*T_c_* is related to small creep strain rates of stages I and II. As a result, the *W_c_* energy is low. On the other hand, in the (f-c) program, the creep load causes a significant increase in the elongation (stages II and III), and as a result, the creep strain energy increases significantly. The opposite situation occurs for the energy *W_f_* which, in the (c-f) test is twice as large as the plastic strain energy dissipated in the (f-c) program.

### 3.5. Accumulation of Damage

The paper presents the experimental verification of the linear damage summation model under the conditions of simultaneous creep and fatigue [7]. The verification was carried out with the use of the durability results, as well as the energy values summarized in Table 1 and Figure 8. Total damage, $D_{t}$, was calculated from relation (6). Table 2 contains the damage values resulting from the fatigue load ($D_{f}$), creep ($D_{c}$), and the total damage $D_{t}$.

**Figure 12:**
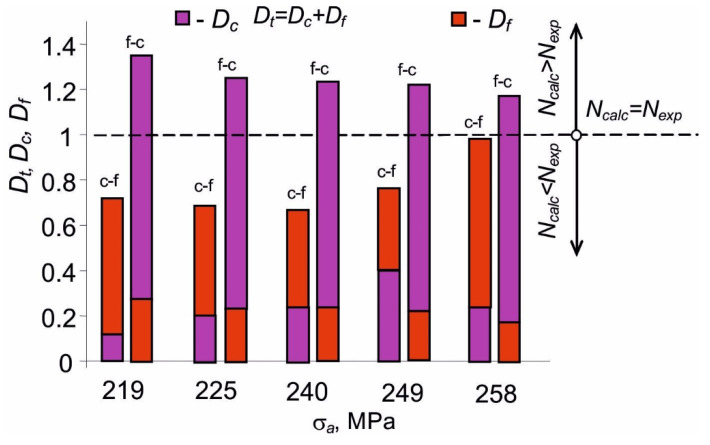
Damage observed during the implementation of programs (c-f) and (f-c).

A comparative analysis of the total damage levels and its components listed in Table 2 reveals that, according to the summation hypothesis (6), the fatigue loads have a dominant effect on the durability of the samples during the (c-f) program variant ($D_{f}>D_{c}$), while creep damage was larger for the (f-c) variant ($D_{c}>D_{f}$). To better illustrate the values of total damage $D_{t}$ and its fractions $D_{f}$ and $D_{c}$, the results are plotted in Figure 12.

The levels of total damage $D_{t}$ different from unity confirm the influence of the sequence of the type of load on durability. The damage values also indicate that the linear model of damage summation under conditions of alternating creep and fatigue load results in qualitatively different lifetime predictions, depending on the load sequence. In the case of the (f-c) program, the durability obtained from simplified calculations is lower than that obtained from tests ($D_{t}$ > 1), so the lifetime predictions are underestimated. However, under the conditions of the (c-f) load sequence, the durability from calculations is higher than that from the tests ($D_{t}$ < 1), so it is overestimated.

## 4. Conclusions

The creep periods that take place alternating with the fatigue load reduce the fatigue life. The sequence of events in the load program has an influence on the durability under the conditions of cyclic load and creep. The cyclic loading preceding creep results in obtaining durability higher than the variant in which creep precedes the variable loads. In light of the above results, it can be concluded that the creep failure and the fatigue failure are not independent. The results confirm the observations presented in [25], that short cracks appear during the fatigue load, and their intensity is higher if the initial creep loading is applied to austenitic stainless steel analyzed in [25]. Disregarding the damage caused by creep in durability calculations may lead to significantly erroneous results. 

The linear damage summation model is insensitive to the sequence of events in the load program. This may result in significant differences between the results of simulations and experimental tests [26,27]. 

In the present paper, only simple load programs were considered. To generalize the conclusions about the influence of creep on the durability of structural elements subjected to fatigue and creep loading sequence, it is necessary to perform tests with the use of complex load programs in which the variable parameter in the test will be creep time τ.

During the tests described in the presented work, the load programs were used in which constant and cyclic loads occurred independently. For this reason, the verification of the linear damage summation hypothesis was limited. To investigate the problem of fatigue in the conditions of simultaneous creep and fatigue, additional experimental tests are required. It is necessary to extend the scope of tests so that each load cycle will be characterized by the presence of both constant and variable loads. An example of such research is described, inter alia, in [4], in terms of deformation. Progress in low-cycle fatigue research, especially in the field of testing machine control abilities, opens up a wide range of possibilities for describing the low-cycle fatigue process in terms of energy.

## Figures and Tables

**Table 1:** Total energy $W_{t}$ and its components depending on the load program.

$\sigma_{a}\;\mathrm{MPa}$	Energy MJ/m^3^							
	Load Variant (c-f)				Load Variant (f-c)			
	$W_{c}$	$W_{f}$	$W_{t}$	N	$W_{f}$	$W_{c}$	$W_{t}$	N
219	7.24	4416.6	4423.8	6116	2154.1	54.29	2208.3	9991
225	10.23	2899.8	2910.0	2046	1420.2	51.68	1471.7	5858
240	11.81	2480.3	2358.1	1743	1022.7	51.36	1047.3	3163
249	19.91	2040.9	2160.4	939	688.5	50.17	738.7	1327
258	10.88	1726.6	1737.5	639	444.1	51.5	495.6	795

**Table 2:** Results of verification of the linear summation hypothesis.

$\sigma_{a}\;\mathrm{MPa}$	$D_{f}$		$D_{c}$		$D_{t}$	
	f-c	c-f	f-c	c-f	f-c	c-f
219	0.27	0.56	1.08	0.14	1.35	0.71
225	0.23	0.48	1.02	0.20	1.25	0.69
240	0.22	0.41	1.01	0.24	1.23	0.64
249	0.21	0.36	1	0.40	1.21	0.76
258	0.17	0.68	1	0.22	1.17	0.90

## Data Availability

Data is contained within the article.
